# A Descriptive Pilot Study of Endothelial Transcriptomic Responses to Extended Lactate Exposure In Vitro

**DOI:** 10.3390/biology15130998

**Published:** 2026-06-25

**Authors:** Daniel Conde, Gabriel Ibarra-Mejía, Manuel Gomez, Alvaro N. Gurovich

**Affiliations:** 1 Clinical Applied Physiology (CAPh) Lab, The University of Texas at El Paso, El Paso, TX 79968, USA; mgomez26@utep.edu (M.G.); agurovich@utep.edu (A.N.G.); 2 Department of Physical Therapy and Movement Sciences, The University of Texas at El Paso, El Paso, TX 79968, USA; 3 Department of Public Health Sciences, The University of Texas at El Paso, El Paso, TX 79968, USA; gabmejia@utep.edu; 4 Interdisciplinary Health Sciences Ph.D. Program, The University of Texas at El Paso, El Paso, TX 79968, USA

**Keywords:** endothelium, glycocalyx, lactate, genes, next-generation sequencing

## Abstract

Lactate is increasingly recognized as a signaling molecule capable of modulating gene expression and vascular function. This descriptive pilot study investigated the effects of different lactate concentrations (0 mM, 10 mM, 20 mM, and 30 mM) and exposure times (1 h, 3 h, and 6 h) on the transcriptomic responses of human umbilical vein endothelial cells (HUVECs). Using next-generation RNA sequencing, an unbiased genome-wide analysis was performed, followed by focused examination of genes relevant to endothelial biology, calcium signaling, and glycocalyx integrity. Results showed that there was no statistically significant effect of lactate concentration on the expression of the examined genes. In contrast, prolonged incubation time was associated with differential expression of *KLF2*, *KLF4*, *FOXO1*, *CD34*, and *VCAM1*. These findings suggest that exposure time, rather than lactate concentration, may be associated with endothelial gene expression patterns under static conditions. This exploratory pilot study provides preliminary transcriptomic observations and highlights the need for further mechanistic investigations including functional and protein-level analyses.

## Simple Summary

Lactate is a molecule produced in the body during intense exercise or illness. While previously considered a waste byproduct, we now know that it can influence cellular behavior. In this descriptive pilot study, we assessed the influence of different lactate concentrations and different exposure times on endothelial cell gene expression using a human umbilical vein endothelial cell (HUVEC) culture model. The results suggest that exposure time, rather than lactate concentration, was associated with changes in endothelial gene expression, including decreased expression of genes associated with endothelial health and increased expression of genes associated with inflammation.

## 1. Introduction

Lactate, traditionally considered a metabolic byproduct of anaerobic glycolysis, is now recognized as a metabolically active molecule involved in cellular signaling, gene regulation, and vascular homeostasis. Recent studies have shown that lactate can influence gene expression through mechanisms including histone lactylation, an epigenetic modification linked with the regulation of genes associated with inflammation, metabolism, and endothelial function [1,2,3,4]. In the vascular system, endothelial cells (ECs) are critical regulators of the hemodynamic balance, barrier integrity, and immune cell signaling. The dysfunction of ECs contributes to the development of cardiovascular pathologies and inflammatory diseases [5,6,7]. The endothelial glycocalyx is a complex layer of proteoglycans and glycoproteins that functions as a mechanosensory and a molecular barrier that modulates vascular permeability, leukocyte adhesion, and shear stress-induced signaling [8,9,10,11]. Recent evidence suggests that alterations in cellular metabolism, including changes in circulating lactate concentrations, may influence the endothelium and glycocalyx integrity, highlighting a potential link between metabolism and vascular function [8,12,13].

Previously considered a byproduct of oxygen-limited metabolism, lactate is now recognized as one of the most abundant carriers of carbon atoms, produced after reduction of pyruvate by nicotinamide adenine dinucleotide (NADH) [14]. Lactate has multiple physiological functions, including acting as an energy substrate for skeletal muscle, cardiac muscle, and the brain; a precursor of gluconeogenesis; a carbon shuttle between tissues; and a signaling molecule capable of regulating gene expression [15]. Under normal physiological conditions, lactate is produced and used by multiple tissues, with the liver serving as a major site of lactate clearance through glucose and glycogen synthesis [15,16,17,18,19,20]. In abnormal cases, including tissue hypoxia, disease, toxin exposure, drug effects, or inherited metabolic disorders, lactate accumulates and causes hyperlactatemia (lactate > 5 mmol/L) [21,22].

Elevated levels of lactate have been observed in a variety of physiological and pathological conditions. During wound healing and tumor-associated angiogenesis, local lactate concentrations can reach 10–15 mM and have been associated with increased vascular endothelial growth factor (VEGF) signaling [23]. Similarly, patients with severe sepsis often have elevated circulating concentrations of lactate (>4 mmol/L) accompanied by markers of endothelial injury [24]. During high-intensity, long-duration exercise, blood lactate concentrations can rise significantly and remain elevated for extended periods. For example, high-intensity interval sprint training can produce average lactate concentrations of approximately 12.7 mmol/L after 10 sprints [25], while marathon running can produce lactate concentrations up to 10 mmol/L after 10 km and remain elevated until the race is completed [26]. Ultramarathon running can also increase lactate concentrations to 3.07 mml/L, and they can remain elevated for an extended period of time [27].

Despite growing evidence that lactate is a signaling molecule, its influence on endothelial gene expression remains incompletely characterized. Previous studies have reported that exposure of human endothelial cells to elevated lactate concentrations can alter the expression of transforming growth factor beta 1 (TGF-β1) [28]. However, a comprehensive understanding of transcriptomic responses to varying lactate concentrations and exposure time is limited. Advances in next-generation sequencing (NGS) allow for a comprehensive, genome-wide characterization of transcriptional response under different experimental conditions [28,29,30].

Therefore, the purpose of this descriptive pilot study was to explore transcriptomic changes in human endothelial cells exposed to different lactate concentrations and incubation times using next-generation sequencing (NGS). Differential expression analysis was performed across the entire transcriptome, followed by focused examination of genes relevant to endothelial function, calcium signaling, oxidative stress, angiogenesis, and glycocalyx biology. This exploratory approach was intended to generate preliminary data that may serve as the basis for future mechanistic studies.

## 2. Materials and Methods

### 2.1. Cell Culture

One vial containing pooled human umbilical vein endothelial cells (HUVECs; Cell Applications, San Diego, CA, USA) was cultured following our previously described methods [31]. In brief, HUVECs were seeded in a T-75 cell culture flask (Corning Inc., Corning, NY, USA) with MesoEndo cell growth medium (Cell Applications, San Diego, CA, USA) in an incubator at 37 °C with 5% carbon dioxide (CO_2_) until 80% confluency. After reaching the desired confluency, cells were trypsinized and subcultured into six 6-well plates (Corning, Fort Worth, TX, USA), where each well represented a technical replicate for each experimental condition (6 technical replicates per condition). Once the cells reached 80% confluency, they were incubated in media supplemented with 0 mM, 10 mM, 20 mM, and 30 mM of sodium lactate (Sigma-Aldrich, St. Louis, MO, USA), following Hashimoto’s experiments with L6 cells [32] and harvested after 1, 3, and 6 h. Measurement of pH in cell culture supplemented with different concentrations of lactate confirmed that pH remained within the optimal range (7.2–7.4).

### 2.2. RNA Isolation

After incubation in lactate, messenger RNA was isolated from HUVECs using the RNeasy Micro Kit (Qiagen, Germantown, MD, USA). Technical replicates of each condition were pooled and immediately sent to the University of Texas at El Paso (UTEP) Genomic Analysis Core Facility (GACF) for analysis. RNA integrity was assessed, resulting in an RNA Integrity Number equivalent (RINe) score of 10. The average concentration of the samples was 86.65 ± 16.28 ng/µL.

### 2.3. Next-Generation Sequencing

RNA sequencing was used for its high sensitivity and throughput compared to RT-qPCR. RNA samples were sequenced at the University of Texas at El Paso Genomic Analysis Core Facility. Sequencing libraries were prepared using the TruSeq stranded mRNA prep kit (Illumina, San Diego, CA, USA). Sequencing was performed using the Illumina NextSeq 2000 (Illumina, San Diego, CA, USA), generating paired-end reads (2 × 150 bp) with an average sequencing depth of 35–50 million reads per sample.

An unbiased genome-wide RNA-seq dataset was generated. Differential Expression analysis was conducted using DESeq2 (ver. 1.46.0). However, because the focus of this pilot study was endothelium-, calcium-, and glycocalyx-associated genes, we report changes only for a selected set of genes. Table 1 summarizes some of the most relevant genes associated with the endothelium, cellular structure, calcium binding, and glycocalyx. The full list of genes analyzed is provided in the Supplementary File S1. Gene information was summarized from the National Institutes of Health, National Center for Biotechnology Information [33].

### 2.4. Differential Expression Analysis

Gene expression data were provided as normalized counts after library size normalization prior to downstream analysis. Sequencing data was imported into RStudio (Ver. 2026.01.0; R Foundation for Statistical Computing, Vienna, Austria) for analysis.

Differential gene expression analysis were performed using the DESeq2 package in R. Gene expression counts were normalized using the median-of-ratios method implemented in DESeq2 [34]. Changes in gene expression associated with lactate concentration or incubation time were evaluated by fitting a negative binomial generalized linear model. Genes with an adjusted *p*-value < 0.05 and an absolute log2 fold change (|log2FC|) ≥ 1 were considered differentially expressed.

## 3. Results

### 3.1. Gene Expression Influenced by Lactate Concentration

**Figure 1:**
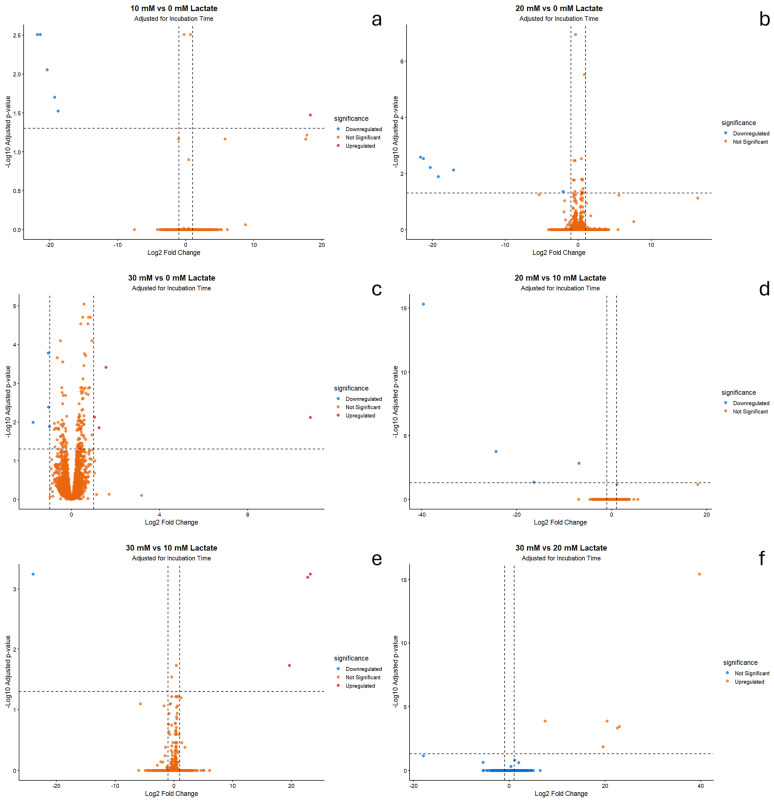
Volcano plots showing genome-wide expression changes associated with different lactate concentrations. (**a**) No differential expression between 10 mM and 0 mM of lactate. (**b**) No differential expression between 20 mM and 0 mM of lactate. (**c**) No differential expression between 30 mM and 0 mM of lactate. (**d**) No differential expression between 20 mM and 10 mM of lactate. (**e**) No differential expression between 30 mM and 10 mM of lactate. (**f**) No differential expression between 30 mM and 20 mM of lactate.

**Figure 2:**
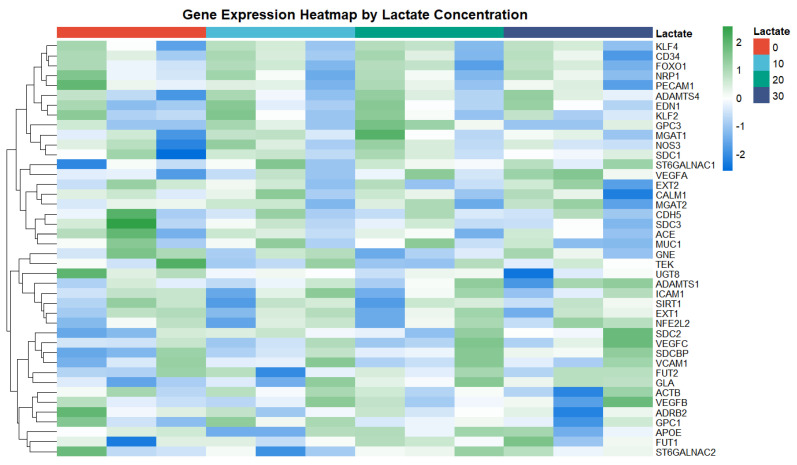
Heat map of target genes across different lactate concentrations. Target genes showed relatively stable expression across all lactate concentrations.

Changes in the expression of genes associated with the endothelium, cellular structure, calcium binding, and the glycocalyx did not differ across lactate concentrations. Volcano plots comparing different lactate concentrations showed that none of the selected target genes met the predefined significance thresholds (adjusted *p* < 0.05). Most genes clustered near the center of the plots with modest fold changes and adjusted *p*-values above the significance threshold, indicating relatively stable expression across lactate concentrations (Figure 1). Heatmap visualization did not show a consistent pattern of upregulation or downregulation among the selected target genes (Figure 2).

### 3.2. Gene Expression Influenced by Incubation Time

**Figure 3:**
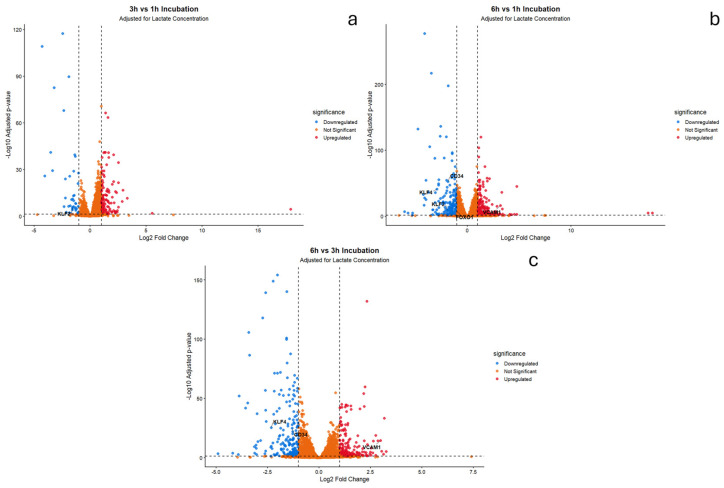
Volcano plots showing genome-wide expression changes associated with different incubation times. (**a**) *KLF2* was differentially expressed 1 h and 3 h (**b**) *KLF2*, *KLF4*, *FOXO1*, and *CD34*, and *VCAM1* were differentially expressed between 1 h and 6 h. (**c**) *KLF4*, *CD34*, and *VCAM1* were differentially expressed between 3 h and 6 h.

**Figure 4:**
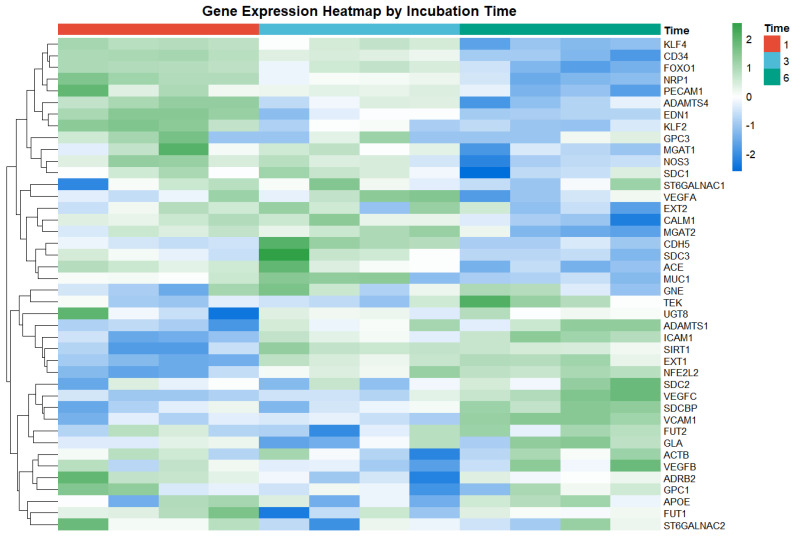
Heat map of target genes across different incubation times. Heatmap visualization suggested modest expression differences among selected target genes after 6 h of incubation.

Genes associated with endothelial function, cellular structure, and the glycocalyx, exhibited differential expression across all incubation times. Volcano indicated that *KLF2* was differentially expressed between 1 h and 3 h; *KLF2*, *KLF4*, *FOXO1*, *CD34*, and *VCAM1* were differentially expressed between 1and 6 h and between 3 h and 6 h. As incubation time increased, several target genes showed greater deviation from basal expression patterns (Figure 3). Heatmap visualizations suggested modest expression differences among the selected genes particularly at 6 h (Figure 4).

## 4. Discussion

The present pilot study explored transcriptomic responses of HUVECs exposed to different lactate concentrations and incubation times. The primary observation was that incubation time, rather than lactate concentration, was associated with differential expression of several genes associated with endothelial biology and the glycocalyx. In contrast, differences in lactate concentrations were not associated with detectable changes in the selected target genes.

Lactate has been considered a byproduct of energy metabolism [35]. However, growing evidence shows that lactate is known to have three key functions: being the main energy substrate in mitochondrial respiration, being the precursor of gluconeogenesis, and functioning as a signaling molecule [2]. In the field of exercise science, lactate is often used as an indicator of exercise intensity, as it correlates with changes in exercise intensity [36,37]. In this pilot study, we exposed HUVECs to 3 lactate concentrations to mimic circulating lactate levels at varying exercise intensities. Across these conditions, there were no transcriptional changes in the selected genes, suggesting that lactate concentration alone may not be the primary factor regulating their expression under the static conditions used in the study.

Similarly, genes associated with the endothelial glycocalyx showed relatively stable expression across different lactate concentrations. The lack of detectable changes may be related to the experimental model. The glycocalyx functions as a mechanosensor that responds to endothelial shear stress and transduces mechanical stimuli into intracellular signaling pathways. Because this pilot study was performed under static conditions, the lack of hemodynamic stimuli may have limited transcriptional responses in glycocalyx-associated genes and reduced the ability to detect potential interactions between lactate signaling and mechanotransduction pathways.

In contrast to lactate concentration, incubation time was associated with changes in the expression of several genes. Longer incubation times were associated with reduced expression of *KLF2*, *KLF4*, *FOXO1*, and *CD34*, and increased expression of *VCAM1*. These findings suggest that the duration of lactate exposure may have a greater association with endothelial transcriptional responses than lactate concentration alone under the conditions studied.

Some of the genes identified in this study have known roles in endothelial homeostasis and inflammation. For example, *KLF2* and *KLF4* are transcription factors involved in the regulation of endothelial phenotype, whereas *VCAM1* is involved in leukocyte-endothelial interactions [38]. Even though the observed expression patterns are consistent with alterations in endothelial inflammation signaling [6], our study did not include functional assessments to determine their downstream effects. Therefore, these observations should be interpreted as preliminary hypothesis-generating results, rather than evidence of endothelial dysfunction or disease-related processes.

It is also important to mention that mRNA abundance does not necessarily reflect protein abundance or biological activity. Many of the genes identified in this pilot study are strongly regulated at the post-transcriptional and post-translational levels. For example, key regulators such as NOS3, KLF2, KLF4, and FOXO1 are influenced by mechanisms including microRNA, mRNA stability, protein phosphorylation, and enzymatic activity, which may affect functional outcomes independently of mRNA abundance [6,38,39,40,41]. Therefore, future studies should incorporate complementary analyses at the protein and functional levels to determine whether the transcriptional changes observed are associated with changes in endothelial function.

Our experiments are not exempt from limitations. We present results from a single pool of HUVECs, which limits the generalizability of the findings. Then, cells were exposed to a constant, static concentration of lactate, which may not fully replicate the dynamic fluctuations in vivo [22]. Furthermore, the lack of osmolarity and cell viability following exposure to high concentrations of lactate limits our ability to distinguish lactate-specific transcriptional responses from potential osmotic stress effects. Finally, the absence of endothelial shear stress may have influenced the expression of mechanosensitive genes, especially those associated with glycocalyx function [42,43]. Future studies should include biological replicates, dynamic flow-based models, protein-level validation, and functional assessments to determine the biological significance of the genes identified in this pilot study.

## 5. Conclusions

In conclusion, this pilot study suggests that incubation time, rather than lactate concentration, is associated with changes in the expression of selected endothelial and glycocalyx-related genes in HUVECs. These findings provide preliminary transcriptomic observations that may help guide future studies exploring the functional significance of prolonged lactate exposure.

## Figures and Tables

**Table 1:** Key genes associated with endothelial health, cellular structure, calcium binding, and glycocalyx structure and function.

**Endothelial Cell-Associated Genes**		
**Gene Name**	**Gene ID**	**Function of the Proteins Encoded**
Krüppel-like transcription factor 2	KLF2	Differentiation and regulation of the functional activity of vascular endothelial cells
Krüppel-like transcription factor 4	KLF4	Promotion of survival by suppressing apoptosis
Intercellular adhesion molecule 1	ICAM1	Regulation of the function of the endothelial and epithelial barrier
Vascular cell adhesion molecule 1	VCAM1	Regulation of inflammation-associated vascular adhesion and migration of leukocytes
Nitric oxide synthase 3	NOS3	Signaling molecule production of nitric oxide
**Cell Structure- and Calcium-Binding-Associated Genes**		
**Gene Name**	**Gene ID**	**Function of the Proteins Encoded**
Actin beta	ACTB	Roles in cell motility, structure, integrity, and signaling
Calmodulin 1	CALM1	Regulation of calcium signaling for the control of cardiac functioning
**Glycocalyx-Associated Genes**		
**Gene Name**	**Gene ID**	**Function of the Proteins Encoded**
Syndecan 1	SDC1	Key roles in glycocalyx structure and integrity, mediation of cell-to-cell and cell-to-matrix interactions, and regulation of growth factor signaling
Syndecan 2	SDC2	Roles in structure, cell-to-matrix interactions, signaling regulation, angiogenesis, and barrier function
CD34 molecule	CD34	Possible role in endothelial barrier function, cell-to-cell interactions, anti-adhesive properties, mechanical protection, and signal transduction
Glypican 1	GPC1	Role in growth factor signaling, cell adhesion, organization of the ECM, and signal transduction
Exostosin glycosyltransferase 1	EXT1	Modification of newly produced enzymes and proteins

## Data Availability

The dataset used in this study is available in online repositories. The files can be accessed on https://github.com/daconde7/Lactate-Exposure-NGS.git (accessed on 15 June 2026).

## Supplementary Materials

The following supporting information can be downloaded at: https://www.mdpi.com/article/10.3390/biology15130998/s1, Table S1a: Summary of the genes associated with endothelial cells analyzed using NGS; Table S1b: Summary of the genes associated with general cell structure and calcium binding analyzed using NGS; Table S1c: Summary of the genes associated with the glycocalyx analyzed using NGS. The full gene list and supporting analyses are available in the Supplemental Materials (File S1). Additional data and analysis files can be accessed at: https://github.com/daconde7/Lactate-Exposure-NGS.git (accessed on 15 June 2026).
