# Supplementary material for: A Descriptive Pilot Study of Endothelial Transcriptomic Responses to Extended Lactate Exposure In Vitro
Source: Biology (Basel). 2026 Jun 25;15(13):998. doi: 10.3390/biology15130998 (PMC13359816; doi:10.3390/biology15130998)
Supplement: Supplementary file 1 [file biology-15-00998-s001.zip › biology-4252622-supplementary.pdf]

Table S1a. Summary of the genes associated with endothelial cells analyzed using NGS.

Endothelial Cell Associated Genes

Gene Name	Gene ID	Function of the Proteins Encoded
Vascular endothelial growth factor A	VEGFA	Induction of proliferation and migration of endothelial cells
Vascular endothelial growth factor B	VEGFB	Regulates the formation of blood vessels
Vascular endothelial growth factor C	VEGFC	Promotes angiogenesis and endothelial cell growth
Nitric oxide synthase 3	NOS3	Signaling molecule production of nitric oxide
Intercellular adhesion molecule 1	ICAM1	Regulation of the function of endothelial and epithelial barrier
Vascular cell adhesion molecule 1	VCAM1	Regulation of inflammation-associated vascular adhesion and migration of leukocytes
Platelet and endothelial cell adhesion molecule 1	PECAM1	Mediation of neutrophil and monocyte transendothelial migration
TEK receptor tyrosine kinase	TEK	Critical roles in maturation and maintenance of the integrity and remodeling of endothelial cells
Neuropilil 1	NRP1	Regulation of multiple signaling pathways in endothelial cells
Krüppel like transcription factor 2	KLF2	Differentiation and regulation of the functional activity of vascular endothelial cells
Krüppel like transcription factor 4	KLF4	Promotion of survival by suppressing apoptosis
Sirtuin 1	SIRT1	Regulation of substrates including eNOS, liver kinase B1, and forkhead box O1
Forkhead box O1	FOXO 1	Regulator of vascular growth the couples metabolic and proliferative activities in endothelial cells
Nuclear factor erythroid 2-derived 2-like 2	NFE2L2	Regulation of the expression of antioxidant proteins to protect against oxidative damage
Apolipoprotein E	APOE	Regulation of endothelial function by modulating basement membrane components
Adrenoreceptor beta 2	ADRB2	Responds to catecholamines to increase ventricular functions and vasodilation
Angiotensin 1 converting enzyme	ACE	A major regulator of blood pressure
Endothelin 1	EDN1	Potent vasoconstrictor

Table S1b. Summary of the genes associated with general cell structure and calcium binding analyzed using NGS.

Cell Structure and Calcium Binding Associated Genes

Gene Name	Gene ID	Function of the Proteins Encoded
Actin beta	ACTB	Roles in cell motility, structure, integrity, and signaling
Calmodulin 1	CALM1	Regulation of calcium signaling for the control of cardiac functioning

Table S1c. Summary of the genes associated with the glycocalyx analyzed using NGS.

Glycocalyx Associated Genes

Gene Name	Gene ID	Function of the Proteins Encoded
Fucosyltransferase 1	FUT1	Role in angiogenesis and ICAM-1 expression
Fucosyltransferase 2	FUT2	Role in angiogenesis
Galactosidase alpha	GLA	Provides the basis for making alpha-galactosidase A to process fatigued cells' components and recycle usable components
Exostosin glycosyltransferase 1	EXT1	Modification of newly produced enzymes and proteins
Exostosin glycosyltransferase 2	EXT2	Involved in the biosynthesis of heparan sulfate, a component of the extra cellular matrix (ECM)
UDP glycosyltransferase 8	UGT8	Role in the modification of the glycosphingolipids
Alpha-1, 3-mannosyl-glycoprotein 2-beta-N-acetylglucosaminyltransferase	MGAT1	Initiation of complex N-linked carbohydrate formation
Alpha-1, 6-mannosyl-glycoprotein 2-beta-N-acetylglucosaminyltransferase	MGAT2	Modification of glycoproteins, affecting the function, cell signaling, and stability
ST6 N-acetylgalactosaminide alpha-2, 6-sialyltransferase 1	ST6GALNAC1	Regulation of cell adhesion, vascular integrity, angiogenesis, and immune response
ST6 N- acetylgalactosaminide alpha-2, 6-sialyltransferase 2	ST6GALNAC2	Similar enzymatic activity as ST6GLANAC1
Mucin 1, cell surface associated	MUC1	Roles in cell protection, cell-to-cell interactions, inflammation, angiogenesis, and signal transduction
Mucin 4, cell surface associated	MUC4	Similar roles as MUC1 and contribution of cell structure
Glucosamine (UDP-N-acetyl)-2-epimerase/N-acetylmannosamine kinase	GNE	Critical role in the biosynthesis of sialic acids, components of the glycocalyx
CD34 molecule	CD34	Possible role in endothelial barrier function, cell-to-cell interactions, anti-adhesive properties, mechanical protection, and signal transduction
Glypican 1	GPC1	Role in growth factor signaling, cell adhesion, organization of the ECM, and signal transduction
Glypican 3	GPC3	Similar role as GPC1
Syndecan 1	SDC1	Key roles in glycocalyx structure and integrity, mediation of cell-to-cell and cell-to-matrix interactions, and regulation of growth factor signaling
Syndecan 2	SDC2	Roles in structure, cell-to-matrix interactions, signaling regulation, angiogenesis, and barrier function
Syndecan 3	SDC3	Similar role as SDC2
Cadherin 5	CDH5	Roles in cell-to-cell adhesion, vascular integrity, and angiogenesis
Syndecan binding protein	SDCBP	Roles in cell adhesion, migration, ECM remodeling and signaling regulation
A disintegrin and metalloproteinase with thrombospondin motifs 1	ADAMTS1	Not directly associated but has roles on vascular homeostasis and endothelial cell biology
A disintegrin and metalloproteinase with thrombospondin motifs 2	ADAMTS2	Indirectly associated with glycocalyx structure, composition, and function
A disintegrin and metalloproteinase with thrombospondin motifs 4	ADAMTS4	May be indirectly associated with the composition and function of the glycocalyx
